# Case Report: Ultrasound-guided hydrodissection targeting the subsynovial connective tissue with dynamic ultrasound assessment in refractory carpal tunnel syndrome: a report of two cases

**DOI:** 10.3389/fmed.2026.1897194

**Published:** 2026-08-28

**Authors:** Daeook Lee, Taeseok Ahn, Myungjin Oh, Jongha Lee, Hyeonjun Woo, Hyunbum Kwon, Changyoung Park, Cheol-Hyun Kim, Jung-Yun Ahn, Jihyun Moon, Dong-Woo Lim

**Affiliations:** 1 SAMSUNG Korean Medicine Clinic, Pohang, Gyeongsangbuk-do, Republic of Korea; 2 VARO Korean Medicine Clinic, Seoul, Republic of Korea; 3 Department of Acupuncture and Moxibustion Medicine, School of Korean Medicine, Pusan National University, Yangsan, Republic of Korea; 4 BAREUNBUBU Korean Medicine Clinic, Naju, Republic of Korea; 5 Oksan Korean Medicine Clinic, Cheongju, Republic of Korea; 6 Samsong Myeongmun Korean Medicine Clinic, Goyang, Republic of Korea; 7 Department of Internal Medicine, College of Korean Medicine, Wonkwang University, Iksan, Republic of Korea; 8 Dasram Cancer Care Hospital, Goyang, Republic of Korea; 9 Department of Diagnostics, College of Korean Medicine, Dongguk University, Goyang, Republic of Korea

**Keywords:** carpal tunnel syndrome, case reports, hydrodissection, subsynovial connective tissue, ultrasonography

## Abstract

Carpal tunnel syndrome is established to be caused by compression of the median nerve. However, recent studies have highlighted fibrosis of the subsynovial connective tissue and consequent restriction of median nerve sliding as a key pathophysiological mechanism. In this case report, we present the results of dynamic ultrasound assessment conducted on two patients with persistent symptoms, with a mixture of polydeoxyribonucleotide (PDRN) 7 mg and 5% dextrose in water (5DW) applied to perform a more extensive ultrasound-guided hydrodissection procedure targeting the subsynovial connective tissue area between the median nerve and the flexor tendons in addition to the conventional area around the median nerve. After treatment, both patients showed a decrease in Boston Carpal Tunnel Questionnaire scores, while dynamic ultrasound evaluation revealed morphological and cross-sectional changes in the median nerve during finger flexion. No adverse events or symptom recurrence were observed during the procedure or throughout the follow-up period. This report highlights an expanded ultrasound-guided hydrodissection approach that targets not only the conventional perineural spaces but also the subsynovial connective tissue-containing intertendinous space in refractory carpal tunnel syndrome. As such, this procedure could represent a non-surgical alternative for patients with carpal tunnel syndrome who do not respond to conventional conservative treatment.

## 1 Introduction

Carpal tunnel syndrome (CTS), a disease caused by compression of the median nerve due to an increase in pressure within the carpal tunnel, is the most common type of peripheral nerve entrapment syndrome (1, 2). Surgical treatment is typically considered for patients with persistent symptoms or who do not respond to conservative treatment; however, there has been growing interest in non-surgical alternatives, as some patients have been reported to show only partial improvement of symptoms after surgery, or to develop complications such as scar pain and nerve injury (3).

Although CTS is classically viewed in terms of static pressure, where increased pressure within the carpal tunnel causes compression of the median nerve, recent pathophysiological studies have increasingly paid attention to the dynamic pathological environment, where interactions between microscopic structures within the carpal tunnel change with finger movement (4). In particular, in the healthy state, the subsynovial connective tissue (SSCT) facilitates sliding between the median nerve and the flexor tendons. However, patients with CTS show non-inflammatory thickening and fibrosis of the SSCT, which has been reported to be a key pathophysiological mechanism restricting median nerve sliding and causing repeated mechanical stress during hand movement due to adhesions in the tissue surrounding the nerve (4, 5). This perspective implies that the connective tissue in the carpal tunnel should be understood not merely as a filler tissue, but instead as a functional ‘microvacuolar system’ that buffers mechanical stress and aids independent sliding between structures (6).

Ultrasound (US) is a non-invasive test instrument that can be used to assess morphological abnormalities, such as edema or flattening of the median nerve, as well as dynamic positional changes and deformations of the nerve with finger movements (7). In particular, US guidance enables the real-time verification of the median nerve and surrounding vascular structures, which can improve the safety and accuracy of procedures (8, 9). Recently, US-guided hydrodissection has been used as a non-surgical intervention; however, the conventional approach only focuses on physically expanding the space compressing the median nerve superiorly and inferiorly (8–10). Although this can relieve compression of the nerve itself, it has limited effect on the aforementioned issue of SSCT adhesions.

The clinical effects of hydrodissection can be influenced not only by expansion of the physical space, but also by the physiological properties of the injected substance. Substances that have recently received interest include polydeoxyribonucleotide (PDRN), which has been proposed to exert effects related to anti-inflammation and tissue recovery, and 5% dextrose in water (5DW), which has been reported to regulate neuropathic pain and improve the microenvironment around the nerve (9, 11, 12). These substances have thus been suggested to relieve physical pressure, while also aiding recovery of the injured nerve and surrounding tissue, and clinical reports using these substances have continually been reported (13).

Based on this context, we report two cases of refractory CTS treated with an expanded US-guided hydrodissection approach that sequentially targeted both the conventional perineural spaces and the SSCT-containing intertendinous space between the flexor tendons. Although US-guided hydrodissection for CTS has been previously reported, the present report focuses on SSCT-oriented target selection and dynamic US assessment of median nerve mobility and deformation before and after treatment. This approach provides a pathophysiology-oriented framework for considering the median nerve sliding environment as a therapeutic target in refractory CTS.

## 2 Case presentation

### 2.1 Patient information

Case 1 was a 56-year-old woman who had complained of numbness in the 1st to 4th digits of the right hand starting approximately 2 years prior, accompanied by tingling and heat that were worse at night. The patient experienced feelings of swelling and stiffness in their hand in the morning after waking, and had received conservative treatment, including physiotherapy and medication at another hospital, but was advised to undergo surgery when these treatment achieved no improvement. The patient is currently a homemaker, but previously had jobs that required repetitive use of the hands. The patient had diabetes but no family history of CTS.

Case 2 was a 59-year-old woman who complained of numbness and decreased sensation in the 1st to 3rd digits of the right hand starting 1 year prior. She had experienced feelings of swelling and stiffness in the hand when active, and had previously received conservative treatment, including injections, at another hospital; however, improvements were minimal. The patient had no underlying diseases or any relevant family history, and was currently working in a job requiring repetitive use of the hands.

This case report adheres to the CARE guidelines, and was approved by the Wonkwang University institutional review board (WKIRB 202601-007). Written consent was obtained from both patients.

### 2.2 Clinical findings

On physical examination at the time of their visit, both patients showed a positive Phalen's test and Tinel's sign, but no atrophy of the thenar eminence was observed. Neither patient reported symptoms suggestive of cervical radiculopathy, such as neck-related radiating pain, and neither patient complained of motor weakness.

### 2.3 Timeline

**Figure 1:**
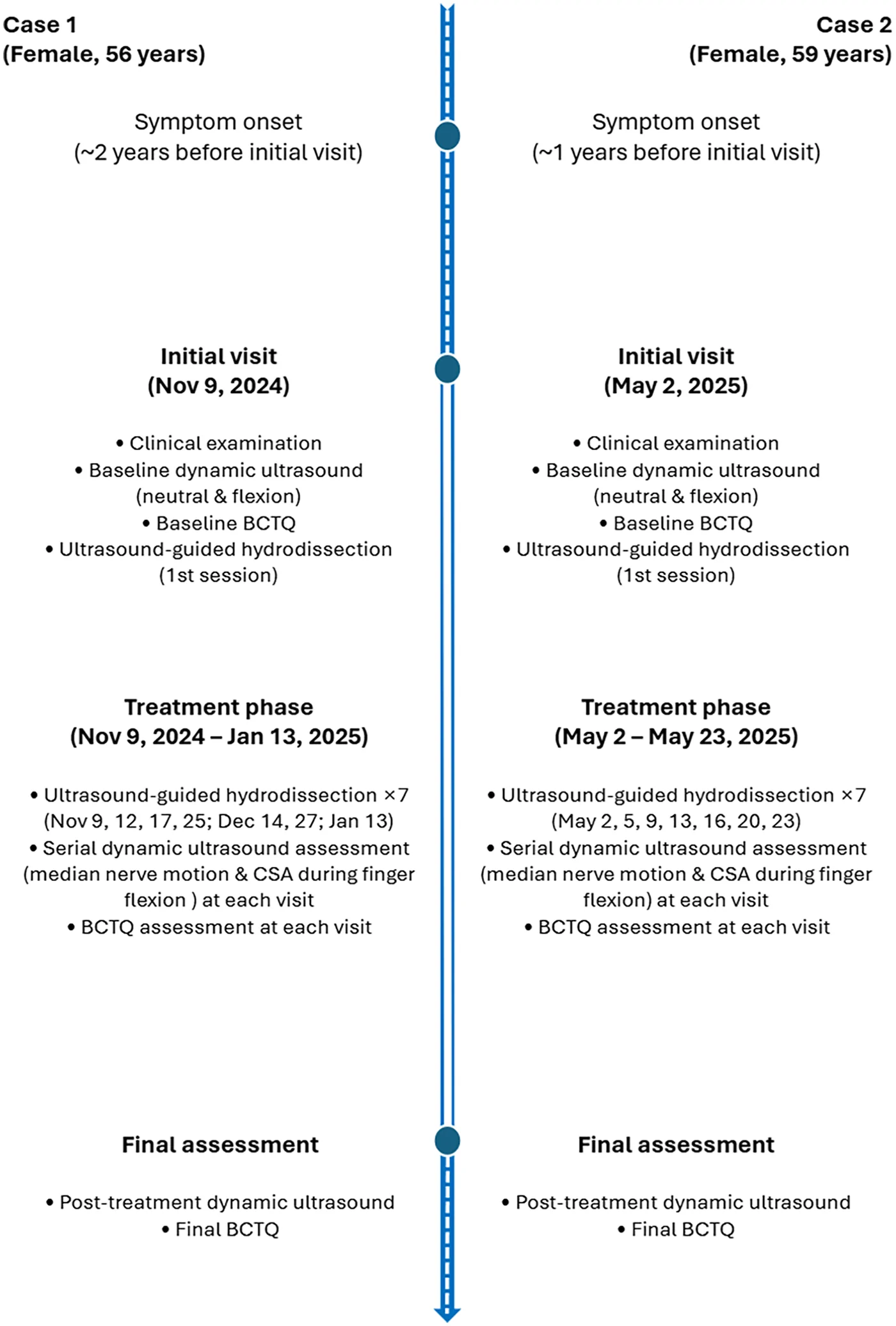
Clinical timeline of the treatment and assessments in two cases of carpal tunnel syndrome. The left and right panels show Cases 1 and 2, respectively. Symptom onset is presented relative to the initial visit. At the initial visit, clinical examination, baseline dynamic ultrasound assessment (neutral and finger flexion positions), and baseline Boston Carpal Tunnel Questionnaire (BCTQ) assessment were conducted, followed by the first session of ultrasound-guided hydrodissection. During the treatment phase, a total of seven ultrasound-guided hydrodissection sessions were conducted (Case 1: Nov 9, 12, 17, 25; Dec 14, 27; Jan 13 and Case 2: May 2, 5, 9, 13, 16, 20, 23), with serial dynamic ultrasound assessment (median nerve motion and cross-sectional area during finger flexion) and BCTQ assessment conducted at each visit. After completion of treatment, post-treatment dynamic ultrasound assessment and final BCTQ assessment were performed. BCTQ, Boston Carpal Tunnel Questionnaire; CSA, cross-sectional area.

Figure 1 presents the timeline of symptom onset, initial visit and subsequent treatment, dynamic US assessment, and assessment of clinical symptoms in both cases.

### 2.4 Diagnostic assessment

The patients were diagnosed based on the combination of clinical symptoms, physical tests, and US examination. To ensure the objectivity and accuracy of US diagnosis and the procedure, all tests were performed by a single trained Korean medicine doctor with at least 15 years of clinical experience and an Alliance for Physician Certification & Advancement (APCA) Registered in Musculoskeletal Sonography (RMSK) qualification. The imaging apparatus used was a GE LOGIQ Fortis and ML 6-15 linear transducer (GE HealthCare, Seongnam, Republic of Korea). The US probe was initially positioned at the proximal wrist crease, between the tendons of the palmaris longus and flexor carpi radialis, and subsequently moved distally across the entire carpal tunnel to the distal margin.

In static US examination, the median nerve was assessed in the longitudinal and transverse directions. In the longitudinal scan, the median nerve notch sign was examined, while in the transverse scan, the cross-sectional area (CSA) of the median nerve was measured at the lunate level, where nerve edema can be readily observed. If the measured CSA was >10.5 mm^2^, this was interpreted as an abnormal radiological finding (14).

**Figure 2:**
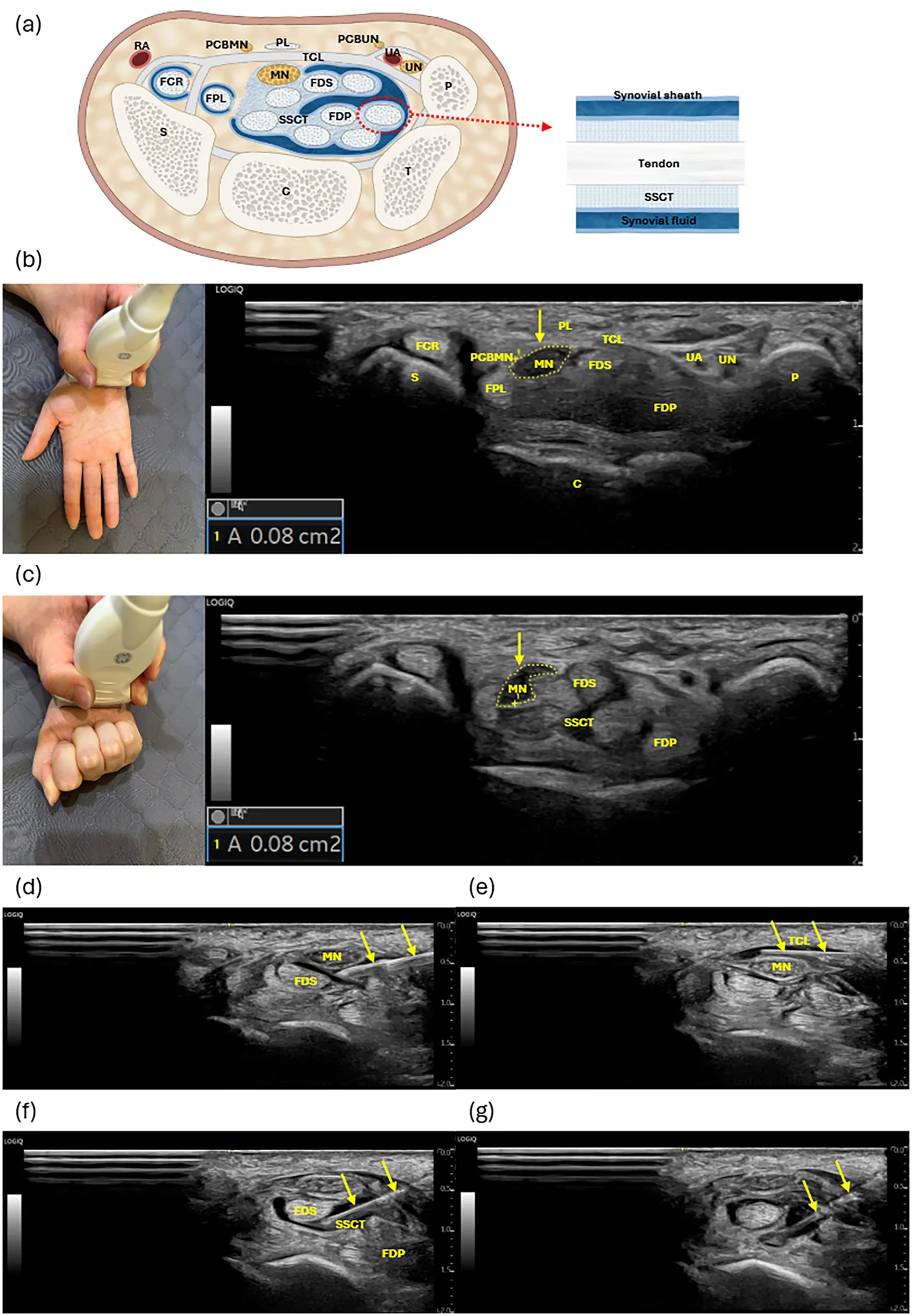
Schematic illustration of the targeted anatomy, reference dynamic ultrasound images, and ultrasound-guided hydrodissection technique for carpal tunnel syndrome. **(a)** Schematic cross-sectional illustration of the proximal carpal tunnel showing the relative positions of the median nerve, flexor tendons, transverse carpal ligament (TCL), and the subsynovial connective tissue (SSCT)-containing intertendinous space targeted during hydrodissection. The inset illustrates the layered relationship among the synovial sheath, SSCT, and the flexor tendons. **(b)** Probe placement and transverse ultrasound image of the proximal carpal tunnel in an asymptomatic normal subject, with the fingers placed in the neutral position. **(c)** Probe placement and transverse ultrasound image during finger flexion in the same asymptomatic normal subject, showing dorsal displacement of the median nerve without obvious flattening or reduction in cross-sectional area. **(d)** Inferior perineural hydrodissection performed beneath the median nerve. **(e)** Superior perineural hydrodissection performed between the transverse carpal ligament and the median nerve. **(f,g)** Hydrodissection of the intertendinous space including the SSCT between the flexor tendons. C, capitate; FCR, flexor carpi radialis tendon; FDP, flexor digitorum profundus tendons; FDS, flexor digitorum superficialis tendons; FPL, flexor pollicis longus tendon; MN, median nerve; P, pisiform; PCBMN, palmar cutaneous branch of the median nerve; PCBUN, palmar cutaneous branch of the ulnar nerve; PL, palmaris longus tendon; RA, radial artery; S, scaphoid; SSCT, subsynovial connective tissue; T, triquetrum; TCL, transverse carpal ligament; UA, ulnar artery; UN, ulnar nerve.

Figure 2a presents a diagram showing the relative positions of the median nerve, flexor tendons, and SSCT, which were the anatomical targets for the procedure, as well as the space target in this case study. To prevent median nerve or PCBMN injury, an in-plane approach was used via an ulnar approach, and the procedure was performed under US guidance.

Dynamic US assessment was conducted to assess median nerve compression and sliding within the carpal tunnel. Finger flexion was performed actively by the patients with the wrist maintained in a neutral position. Representative transverse images obtained at the neutral and finger-flexion positions were used to evaluate median nerve movement, flattening, and changes in CSA. This examination was performed at the scaphoid and pisiform level, corresponding to the proximal carpal tunnel. Movement of the median nerve and changes in the CSA were observed in real time, while the patient slowly flexed her fingers. Figures 2b,c present reference images from a symptomless healthy individual acquired using the same method.

Sensation in the proximal palm, which is innervated by the palmar cutaneous branch of the median nerve (PCBMN), was preserved in both cases. In addition to the carpal tunnel assessment, ultrasound tracking was performed along the proximal course of the median nerve at common sites of entrapment, and no focal swelling, compression, mass-like lesion, or other abnormal ultrasonographic findings were observed outside the carpal tunnel region. Together with the absence of neck-related radiating pain or motor weakness, these findings supported localization of the lesion to the carpal tunnel. However, electrodiagnostic studies were not performed; therefore, objective severity grading, differentiation between demyelinating and axonal involvement, and definitive exclusion of cervical radiculopathy or proximal median neuropathy were limited. In summary, both cases were clinically diagnosed as CTS based on symptoms, physical examination findings, and static and dynamic US findings.

### 2.5 Therapeutic intervention

In both cases, patients were treated using US-guided hydrodissection. The injectant was prepared by mixing 2 mL of solution containing 7 mg PDRN and 3 mL of 5DW, and was injected using a 5 mL syringe with a 38 mm 27-gauge needle. The procedure was performed 7 times, at a frequency of 1–2 times/week (Figure 1). The seven-session protocol was based on institutional clinical practice and clinical experience rather than on a standardized evidence-based regimen. During the treatment period, the patients only underwent US-guided hydrodissection and did not receive any other CTS-related interventions (e.g., acupuncture, moxibustion, cupping, drug therapy, physiotherapy, injections, or surgery) at our hospital or any other institution.

The injection was made in three stages:

Stage 1 (inferior to the median nerve): The needle was advanced in a ‘bevel up’ position inferior to the median nerve. The drug was injected along the epineurium to achieve hydrodissection of the connective tissue located between the median nerve and the flexor tendons (Figure 2d).

Stage 2 (superior to the median nerve): The needle was advanced in a ‘bevel down’ position superior to the median nerve, and the drug was injected around the epineurium to achieve hydrodissection of the space between the transverse carpal ligament and the median nerve (Figure 2e).

Stage 3 (SSCT between the flexor tendons): The needle was advanced again in a ‘bevel up’ position inferior to the median nerve to achieve hydrodissection of the SSCT between the flexor tendons (Figures 2f,g).

No adverse events or complications, including nerve injury, were observed during the procedures in both cases.

### 2.6 Case 1 – ultrasound findings and clinical outcomes

**Figure 3:**
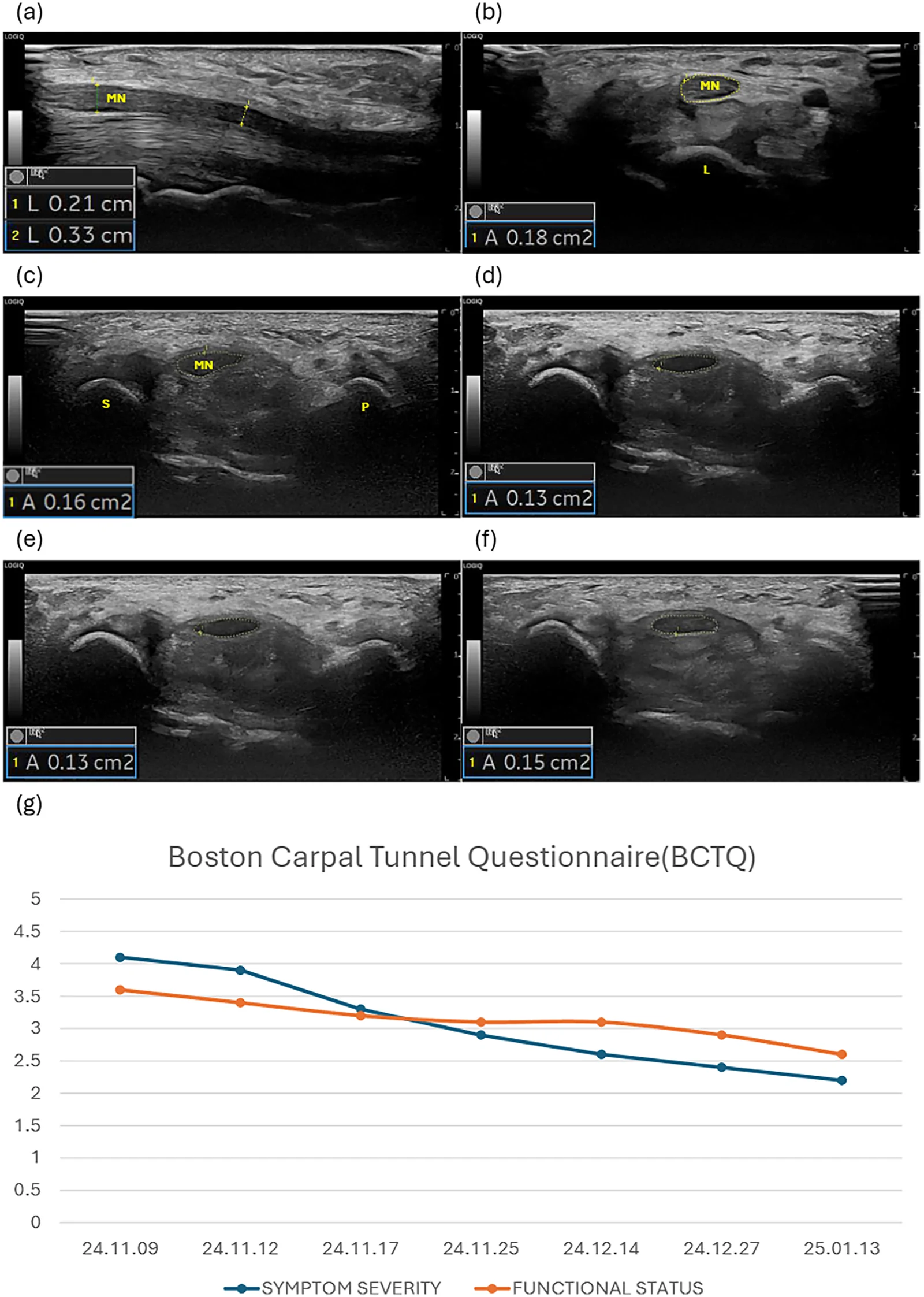
Ultrasound findings and clinical outcomes in case 1. **(a)** Longitudinal ultrasound image of the median nerve showing a notch sign. **(b)** Transverse ultrasound image at the lunate level demonstrating an increased cross-sectional area (CSA) of the median nerve (18 mm^2^). **(c)** Transverse ultrasound image at the proximal carpal tunnel, with the fingers in a neutral position showing the location and CSA of the median nerve. **(d)** Transverse ultrasound image taken during finger flexion demonstrating ventral displacement and flattening of the median nerve with a reduced CSA (16 mm^2^ → 13 mm^2^). **(e)** Pre-treatment ultrasound image showing the CSA of the median nerve measured at the proximal carpal tunnel (13 mm^2^). **(f)** Post-treatment ultrasound image demonstrating an increased CSA (15 mm^2^) and improved morphology of the median nerve after ultrasound-guided hydrodissection. **(g)** Changes in Boston Carpal Tunnel Questionnaire (BCTQ) scores during the treatment period, showing progressive improvement in symptom severity and functional status. CSA, cross-sectional area; L, lunate; MN, median nerve; P, pisiform; S, scaphoid.

In the longitudinal scan before treatment, a positive median nerve notch sign was observed (Figure 3a), and in the transverse scan, the peak median nerve CSA measured at the level of the lunate was 18 mm^2^ (Figure 3b). In dynamic US assessment at the scaphoid and pisiform level, corresponding to the proximal inlet of the carpal tunnel, during finger flexion, the median nerve moved ventrally between the flexor tendons and the transverse carpal ligament, and the CSA decreased from 16 mm^2^ to 13 mm^2^ as the median nerve flattened (Figures 3c,d). Conversely, in dynamic US assessment after hydrodissection, the median nerve was confirmed to slide freely without compression from surrounding tissues. Before the procedure there had been some decrease in CSA during finger flexion as the median nerve was flattened, but after the procedure, there was sufficient space for nerve movement, and the CSA remained constant even during flexion (Figures 3e,f).

Treatment response was assessed using the Boston Carpal Tunnel Questionnaire (BCTQ). This assessment revealed clear decreases in scores for both symptom severity (baseline: 4.1 points, post-treatment: 2.2 points) and functional status (baseline: 3.6 points, post-treatment: 2.6 points; Figure 3g). The patient completed all seven planned treatment sessions, indicating good treatment compliance. These clinical improvements were stable throughout follow-up monitoring, and there were no treatment-related adverse events. Even 12 weeks following completion of treatment, there was no recurrence, and the clinical improvement was maintained.

### 2.7 Case 2 – ultrasound findings and clinical outcomes

**Figure 4:**
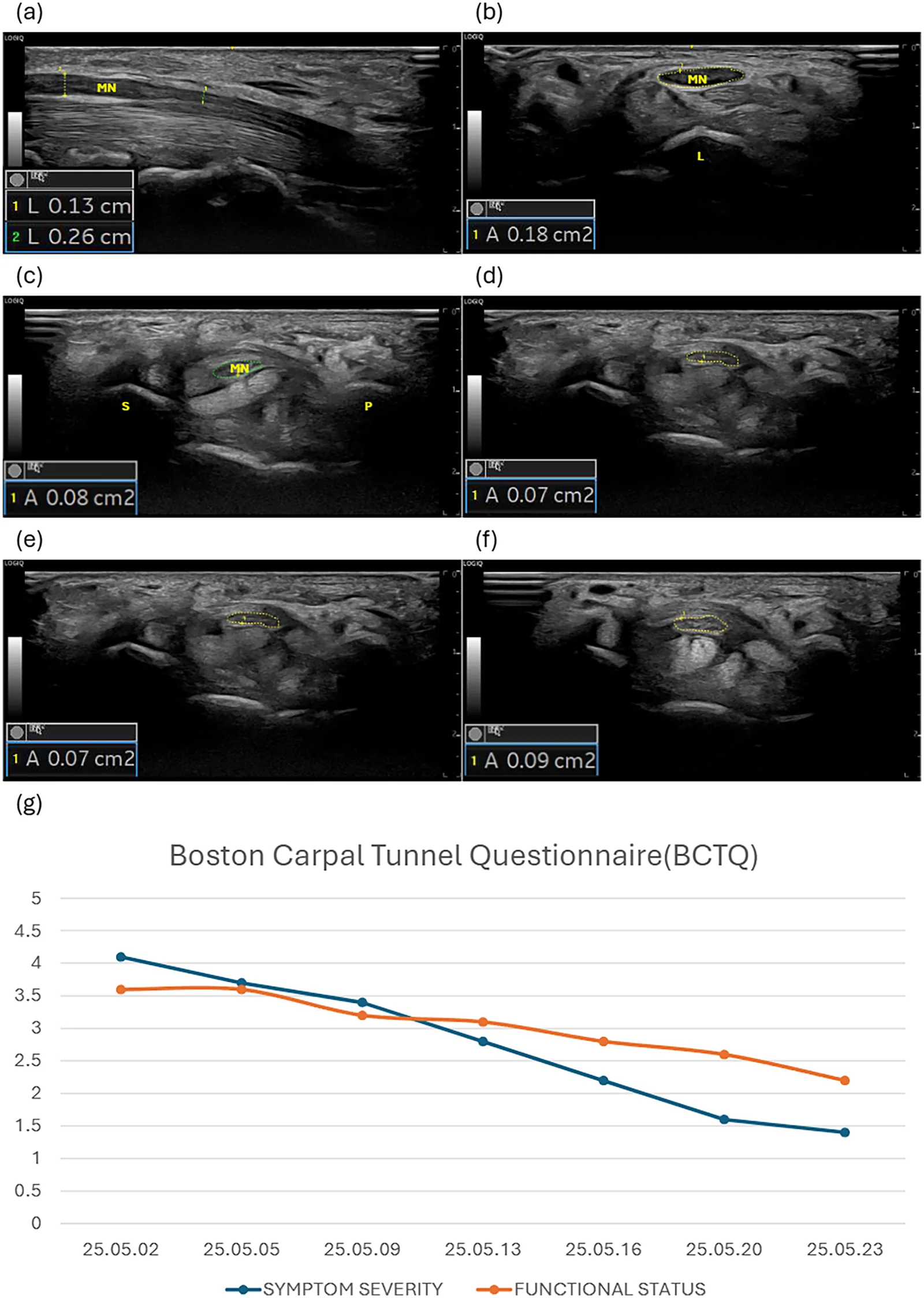
Ultrasound findings and clinical outcomes in case 2. **(a)** Longitudinal ultrasound image of the median nerve showing a notch sign. **(b)** Transverse ultrasound image taken at the lunate level demonstrating an increased cross-sectional area (CSA) of the median nerve (18 mm^2^). **(c)** Transverse ultrasound image at the proximal carpal tunnel with the fingers in a neutral position showing the location and CSA of the median nerve. **(d)** Transverse ultrasound image during finger flexion demonstrating ventral displacement and flattening of the median nerve with a reduced CSA (8 mm^2^ → 7 mm^2^). **(e)** Pre-treatment ultrasound image showing the CSA of the median nerve measured at the proximal carpal tunnel (7 mm^2^). **(f)** Post-treatment ultrasound image demonstrating an increased CSA (9 mm^2^) and improved morphology of the median nerve following ultrasound-guided hydrodissection. **(g)** Changes in Boston Carpal Tunnel Questionnaire (BCTQ) scores during the treatment period, showing progressive improvements in symptom severity and functional status. CSA, cross-sectional area; L, lunate; MN, median nerve; P, pisiform; S, scaphoid.

A positive median nerve notch sign was observed in the pre-treatment longitudinal scan (Figure 4a), while in the transverse scan, the peak median nerve CSA measured at the level of the lunate was 18 mm^2^ (Figure 4b).

In dynamic US assessment at the level of the proximal carpal tunnel, ventral displacement and flattening of the median nerve were observed during finger flexion, with the CSA decreasing from 8 mm^2^ to 7 mm^2^ (Figures 4c,d).

In the follow-up dynamic US assessment after hydrodissection, the decrease in CSA that was previously observed during finger flexion had been ameliorated. With the decrease in mechanical stress, the median nerve maintained a stable shape and position among the surrounding tissues (Figures 4e,f).

Treatment response was assessed using the BCTQ, which demonstrated marked reductions in both symptom severity scores (baseline: 4.1 points, post-treatment: 1.4 points) and functional status scores (baseline: 3.6 points, post-treatment: 2.2 points; Figure 4g). The patient completed all seven planned treatment sessions, demonstrating good treatment compliance. These clinical improvements remained stable throughout the follow-up period, and there were no treatment-related adverse events. Furthermore, no recurrence was observed at the 23-week follow-up after treatment completion, and the clinical improvement was maintained.

### 2.8 Patient perspective

Before the procedure, Case 1 expressed difficulty sleeping due to feelings of heat and numbness at night. They were advised to undergo surgery, but preferred to receive non-surgical treatment. After the procedure, the patient reported decreased nocturnal heat sensation and numbness, and improved sleep quality. Case 2 also reported less discomfort during finger flexion, less stiffness in the morning, and easier use of the hand in daily living. Both patients expressed satisfaction at having achieved symptom relief after the non-surgical intervention.

## 3 Discussion

CTS is a common peripheral nerve entrapment lesion which most commonly develops in patients in their 40s to 60s (1). For patients in whom symptoms persist for several months, the response to conservative treatment can be limited (15). Surgical treatment is considered in some patients, but due to reports of postoperative complications and residual symptoms, there is consistent interest in non-surgical interventions (3). Within this clinical context, in the present case report we explore the potential of a non-surgical intervention in patients with persistent, long-term symptoms.

The anatomical structure of the carpal tunnel is complex; the median nerve and flexor tendons run through a space formed by the carpal bones and the transverse carpal ligament, and the intermediate space is connected by SSCT. In previous studies, structural changes in the SSCT were shown to interfere with free sliding of the median nerve, which can affect the mechanical interactions between the nerve and surrounding tissue within the carpal tunnel (4, 5). In this situation, ventral displacement, deformation, and reduced CSA of the median nerve have been reported during finger flexion (4, 5).

A CSA cut-off of 10.5 mm^2^ was applied as the diagnostic criterion based on a recent meta-analysis demonstrating its reliability in Asian populations (14). We acknowledge that electrodiagnostic studies, including nerve conduction studies and electromyography when indicated, remain important reference standards for confirming CTS, grading disease severity, distinguishing demyelinating from axonal involvement, and excluding other conditions such as cervical radiculopathy or proximal median neuropathy. In the present cases, EDX was not performed because the patients presented for ultrasound-guided non-surgical intervention after persistent symptoms despite previous conservative management, and additional electrodiagnostic testing was not performed because of referral logistics, patient preference, and the clinical workflow of this case report. Therefore, the diagnosis and follow-up assessment were based on clinical symptoms, physical examination findings, and static and dynamic US findings (16, 17).

Unlike conventional hydrodissection, which predominantly targets the perineural space around the median nerve, the present approach sequentially extended the dissection to the intertendinous space within the SSCT. This modification was based on the pathophysiological rationale that SSCT fibrosis and adhesion increase resistance to median nerve sliding during finger movement, thereby contributing to persistent symptoms (4, 5).

The microvacuolar system described by Guimberteau et al. provides a conceptual framework for understanding the SSCT not merely as passive connective tissue, but as a functional sliding environment that buffers shear stress between adjacent structures (6). The expanded hydrodissection approach used herein can accordingly be interpreted as an attempt to restore this sliding environment within the intertendinous space of the carpal tunnel.

The PDRN and 5DW used in this case report were selected not only as a simple dissection media, but also based on their potential to aid tissue recovery and improve the perineural microenvironment. PDRN has been proposed to exert effects related to anti-inflammation and tissue regeneration, and clinical trials in patients with CTS have reported an association with symptom improvement (11). Similarly, 5DW has been shown to be associated with neuropathic pain relief and improvement of the perineural environment when used in hydrodissection (9, 12). In summary, this procedure combines the physiological recovery effects of these drugs with the mechanical hydrodissection effect whereby the pressure of the injectant causes adhesiolysis; this combined effect is believed to have resulted in the significant improvement in patients with severe disease who had been advised to undergo surgery.

Unlike prior studies that have predominantly focused on static nerve morphology, the present cases demonstrated post-treatment improvements in dynamic ultrasonographic findings, including restoration of nerve shape and CSA during finger flexion. These changes may be interpreted as indirect evidence of an improved sliding environment within the carpal tunnel, although direct verification of microstructural SSCT changes was beyond the scope of US imaging alone. Subjective symptom improvement was consistent with objective findings, as reflected by improvements in both subscales of the BCTQ in both cases (18).

This study had several limitations. First, electrodiagnostic studies were not performed. Although the diagnosis was supported by typical clinical symptoms, positive physical examination findings, and static and dynamic US findings, the absence of nerve conduction studies limited objective severity grading and the exclusion of other neurophysiological differential diagnoses. Second, the dynamic US assessment could have been affected by the protocol and the examiner's proficiency. Third, because this was an observational report of a small number of cases, there are limitations in generalizing the treatment effects or investigating pathophysiological causality. The findings cannot establish treatment efficacy, generalizability, or pathophysiological causality. The observed improvements should be interpreted as hypothesis-generating clinical observations rather than definitive evidence of effectiveness. In addition, in this case report, it was not possible to differentiate between the physiological effects of the injected drugs and the mechanical pressure-decreasing effects of the hydrodissection itself, meaning that we cannot conclude whether the observed clinical changes were caused by a specific mechanism. Future prospective studies should include larger sample sizes, standardized dynamic US assessment protocols, electrodiagnostic confirmation, and appropriate control groups to clarify the clinical significance of SSCT-targeted hydrodissection.

## 4 Conclusion

In summary, this exploratory two-case report describes US-guided hydrodissection targeting the perineural spaces and the intertendinous SSCT, combined with dynamic US assessment, in patients with refractory CTS. Although the observed clinical and dynamic US changes are encouraging, they should be interpreted cautiously because of the small number of cases.

## Data Availability

The original contributions presented in the study are included in the article/Supplementary Material, further inquiries can be directed to the corresponding authors.
